# A shift from papillary to reticular fibroblasts enables tumour–stroma interaction and invasion

**DOI:** 10.1038/s41416-018-0024-y

**Published:** 2018-03-19

**Authors:** Marieke Hogervorst, Marion Rietveld, Frank de Gruijl, Abdoelwaheb El Ghalbzouri

**Affiliations:** 1 Department of Dermatology, Leiden University Medical Centre, 2333 ZA Leiden, The Netherlands; 2 Biomimiq, J.H. Oortweg 19, 2333 CH Leiden, The Netherlands

**Keywords:** Cancer microenvironment, Cancer models, Squamous cell carcinoma

## Abstract

**Background:**

Tumour stroma consists of a heterogeneous population of fibroblasts and related mesenchymal cells, collectively dubbed cancer-associated fibroblasts (CAFs). These CAFs are key players in cancer invasion of cutaneous squamous cell carcinoma (SCC). As we have shown earlier, papillary and reticular fibroblasts (Pfs and Rfs, respectively) have distinct effects on epidermal and dermal homeostasis, but their role in cancer invasion and epithelial-to-mesenchymal transition (EMT) remains to be determined.

**Methods:**

We used 3D cultures of human skin equivalents (HSEs) to analyse the effects of Pfs and Rfs on the invasive behaviour of SCC and EMT.

**Results:**

We reveal for the first time the importance of Pfs versus Rfs in SCC invasion and EMT. Cell lines from different stages of SCC showed significantly more extensive invasion into a dermis composed of Rfs than of Pfs. In addition, Rfs-based HSEs showed increased cell activation and stained positive for CAF biomarkers α-SMA and vimentin. Further analysis revealed that invasively growing cancer cells in Rf-HSEs express markers of EMT transition, like SNAIL2, N-cadherin and ZEB1.

**Conclusions:**

Conversely, our results show that Pfs contain cancer cells more within the epidermis. Rfs are clearly predisposed to differentiate into CAFs upon SCC signals, assisting invasion and EMT.

## Introduction

The crosstalk between the different components in the tumour microenvironment is recognised as essential in cancer development and is becoming the main focus in cancer research.^1^ It is known that the tumour stroma is an integral part of cancer development. The basement membrane (BM), immune cells, fibroblasts and extracellular matrix (ECM) surrounding the cancer cells constitute this stroma, which actively contributes to cancer progression.^2^ In cutaneous squamous cell carcinoma (SCC), invasion occurs upon disrupted epidermal homeostasis, resulting from interactions between the epidermal cancer cells and their underlying stroma.^3^ One mechanism that facilitates SCC metastasis is epithelial-to-mesenchymal transition (EMT). In epithelial tumours, EMT is crucial for the migration and invasion of cancer cells.^4^ Herein, a cell loses its epithelial phenotype and tight cell–cell junctions and acquires a mesenchymal phenotype that allows detachment and migration from the primary tumour into the surrounding stroma.^5^ Certain fibroblasts, abundant in the tumour stroma, are associated with SCC progression; they have structural and functional contributions to this process. These so-called cancer-associated fibroblasts (CAFs) produce growth factors and chemokines, which facilitate angiogenesis, tissue invasion and metastasis. In concert with epithelial cancer cells, CAFs have been found responsible for breakdown of BM structures (by release of, e.g., metalloproteinases) and dermal ECM remodelling, thereby facilitating tumour invasion.^6^

The dermis contains a variety of fibroblast exhibiting functional diversity.^7^ The papillary dermis is characterised by a relatively thin ECM combined with a high cell density, whereas the reticular ECM has a very dense network of matrix fibres and a low cell density.^8^ Previous studies from our group have shown that (a) papillary and reticular fibroblasts (Pf and Rf, respectively) exhibit subtype-specific gene expression profiles; (b) differently affect skin homeostasis; and that (c) Pfs can differentiate towards a reticular phenotype by “in vitro ageing” or by transforming growth factor-β activation.^9–11^ In addition, ECM generated by Pfs better supported epidermal longevity compared to reticular-generated ECM.^12^ These findings indicate that Pfs and Rfs generate dissimilar ECMs affecting skin homeostasis. For example, recently it has been shown that initial wound healing is mediated by Rfs, while Pfs play a crucial role in the final stage of this process.^9^ These fibroblast subtypes may also affect skin-ageing processes. With ageing, the papillary dermis gets thinner, while the reticular layer becomes thicker.^8,11^ The loss of Pfs has a profound effect on skin homeostasis as reported both in in vivo and in vitro studies.^8,9^ This could explain why the speed of wound closure reduces with age.^13^ One may speculate that the dissimilar ECMs generated by Pfs and Rfs also affect stroma composition that either promotes or inhibits cancer development. To explore this hypothesis, we examined whether (i) SCC undergoes invasion through EMT, (ii) reticular or papillary-derived fibroblasts are able to promote or inhibit this process and (iii) Rfs differentiate towards CAFs.

To study the clinically highly relevant effect of the fibroblast lineages abundant in the tumour stroma on invasion and early metastasis of cutaneous SCC, we employed two types of in vitro full-thickness skin models (FTMs): one with solely papillary dermis (Pf-FTM) and one with a reticular dermis (Rf-FTM). Since Rfs are different from their papillary counterparts, this causes changes in the dermal and epidermal microenvironment that not only contributes to skin ageing but also may affect the growth and migrating behaviour of SCC. We investigated the behaviour and invasive characteristics of four SCC cell lines in FTMs based on a human fibroblast‐seeded rat‐tail collagen. The cell lines were derived from different stages of SCC. In the analysis of models, we focused on ingrowth, epidermal hyper proliferation, activation and differentiation, all crucial assets of invasive SCC. Furthermore, we unravelled the mechanism causing SCC invasion in vitro, focussing on the effect of fibroblast subpopulations on EMT. In the past decade, a repertoire of EMT biomarkers involved in metastasis of SCC has been described.^14^ These biomarkers were assessed in our in vitro SCC-FTMs. The most important factor in EMT is the reduction of cell–cell adhesion molecule E-cadherin and upregulation of more mesenchymal proteins, such as vimentin, N-cadherin and α-smooth muscle actin (α-SMA).^15^ E-cadherin is, in its turn, repressed by EMT-inducing transcription factors, like TWIST, SNAIL and ZEB.^16–18^ The presented data demonstrate that fibroblast subtypes and the composition of the dermal microenvironment play a crucial role in the invasive behaviour of SCC.

## Materials and methods

### Patient material

SCC biopsies were obtained from the archive of the Leiden University Medical Centre pathology department. The biopsies were either fixed in 4% paraformaldehyde, dehydrated and paraffin embedded or cryopreserved.

### Cell culture

PFs and Rfs were isolated from different dermal layers of surplus skin from female donors (age 31–47 years) undergoing abdominal surgery as described earlier.^8,19^ Briefly, the Pfs originate from a depth of 100–300 μm; for the reticular dermis, a minimum of 700 μm is required. The different dermal layers were separated using a dermatome. Fibroblasts were isolated from the dermis by treatment with collagenase (Invitrogen, Breda, The Netherlands) and dispase II (ratio 1:3 and 3 ml/g dermis) (Roche Diagnostics, Almere, The Netherlands) for 2 h at 37 °C. The cells were subsequently filtered using a 70 μm cell strainer and cultured. Standard fibroblast medium consisted of Dulbecco’s modified Eagle’s medium (DMEM, Gibco/Invitrogen) supplemented with 5% foetal bovine serum (FBS, HyClone/Greiner, Nurtingen, Germany and 10 mg/ml penicillin/streptomycin (Invitrogen).

For isolation of normal human epidermal keratinocytes (NHEKs), epidermis was obtained through overnight incubation of fresh mamma reduction surplus skin with dispase II (Roche Diagnostics, Almere, The Netherlands). NHEKs were isolated from the epidermis through incubation with trypsin at 37 °C for 15 min. After trypsin inactivation, the cells were filtered using a 70 μm cell strainer (BD Biosciences, Breda, The Netherlands) and cultured in keratinocyte medium at 37 °C and 7.3% CO_2_ until subconfluency. Standard keratinocyte medium consisted of DMEM and Ham’s F12 medium in a 3:1 ratio, supplemented with 5% FBS, 0.28 μM hydrocortisone, 1 mM isoproterenol, 0.87 mM insulin (Sigma-Aldrich, Zwijndrecht, The Netherlands) and 10 mg/ml penicillin/streptomycin. All surplus material was obtained in accordance with the Dutch Law on Medical Treatment Agreement.

SCC-12B2 (isolated from a facial SCC on a 60-year-old male kidney transplant patient) was previously kindly provided by Dr J.G. Rheinwald.^20^ SCC12b2 cells were initiated on lethally irradiated murine 3T3 fibroblasts in standard keratinocyte medium at 37 °C and 7.3% CO_2_ until subconfluency. After a few passages, they were cultured further without the feeder cells. The lines MET1, MET2 and MET4 cell lines were kindly provided by Professor C. Proby (Division of Cancer Research, Medical Research Institute, Jacqui Wood Cancer Centre Ninewell Hospital & Medical School, Dundee).^21^ The MET lines were derived from the same patient, but from an invasive (MET1), a recurrent (MET2) and metastatic SCC (MET4). The cells were thawed and cultured in standard keratinocyte medium supplemented with 10 ng/ml epidermal growth factor (EGF) at 37 °C and 7.3% CO_2_ until subconfluency. After a few passages, the EGF concentration was reduced to 2 ng/ml.

### SCC-FTM generation

Rat-tail collagen dermal matrices were constructed as described earlier.^22^ Subsequently, each of the constructs were seeded with 5 × 10^4^ SCC cells (MET1, MET2, MET4 or SCC12b2). For the co-culture, these SCC cells were mixed with NHEKs (1:4) prior to seeding. FTMs were incubated overnight under submerged conditions at 37 °C and 7.3% CO_2_ in standard keratinocyte medium as described above. After 3 days, FBS was reduced to 1%. Two days thereafter, FBS was omitted and human skin equivalents (HSEs) were cultured at the air–liquid interface for 14 days. During this period, HSEs were cultured in keratinocyte medium as described above but without serum and supplemented with 2 M l-serine, 10 mM l-carnitine, 1 μM DL-α-tocopherol-acetate, 50 μM ascorbic acid, 2.4 × 10^−5^ M bovine serum albumin and a free fatty acid supplement that contained 25 μM palmitic acid, 30 μM linoleic acid and 7 μM arachidonic acid (Sigma-Aldrich). Culture medium was refreshed twice a week.

### Morphological and immunohistochemical (IHC) analysis

From the HSEs, one part was snap-frozen in liquid nitrogen while the other part was fixed in 4% paraformaldehyde, dehydrated and paraffin embedded. Global histological analysis was performed on 5 μm sections through haematoxylin and eosin (HE) staining. Immunohistochemical analysis was performed using the streptavidin–biotin–peroxidase system (GE Healthcare, Buckinghamshire, UK), according to the manufacturer’s instructions. Staining was visualised with 3-amino-9-ethylcarbazole, counterstained with haematoxylin and mounted with Kaiser’s glycerine. For antigen retrieval, sections were boiled in citrate buffer (pH 6.0) in an autoclave at 110 °C for 5 min (for ki67, k16, k17, k10 and loricrin) or incubated with 0.025% protease-X (Sigma, Zwijndrecht, The Netherlands) at 37 °C for 40 min (for collagen type IV). For fluorescence staining, sections were labelled with the primary antibody as described above and counterstaining was performed with 4,6-diamidino-2-phenylindole. Primary antibodies are specified in Table 1.

**Table 1:** Primary antibody specifications

Antibody	Clone	Host	Dilution	Source
Collagen type IV	PHM12	Mouse	1:150	Chemicon, Temecula, USA
Ki67	MIB1	Mouse	1:75	Dako, Glostrup, Germany
Keratin 17	CK-E3	Mouse	1:25	Novus Biologicals, Colorado, USA
Keratin 10	DE-K10	Mouse	1:100	Labvision/neomarkers, California, USA
Loricrin	AF62	Rabbit	1:1000	Covance, USA
α-SMA	1A4	Mouse	1:400	Abcam
Laminin332	BM165	Mouse	1:75	Dr. M. Aumailley (Cologne, Germany)
ZEB1		Rabbit	1:666	Atlas antibodies
B-cat	14/Beta-Catenin	Mouse	1:250	BD Bioscience
TGM2	CUB7402	Mouse	1:50	Abcam
PDPN	18H5	Mouse	1:100	Abcam
TNC	EPR4219	Rabbit	1:100	GeneTex

Secondary antibodies included biotinylated goat anti-mouse IgG secondary antibody (Dako, Glostrup, Germany), swine anti-rabbit igG secondary antibody (Dako, Glostrup, Germany), biotinylated goat anti-mouse IgG secondary antibody (Jackson) and goat anti-rabbit Alexa 488 (Invitrogen).

### Quantification of ingrowth

Pictures of the HE staining were converted to greyscale. In the program Image J, boundaries were set manually along the ingrowth areas of the HSEs. Subsequently, the inverse average intensity per pixel was multiplied by the area. Results were expressed as the mean ± SD of the four counts in three different donors (error bars).

### Quantification of proliferation index

To determine the proliferation index of the FTMs cultured with SCC cells, the number of Ki67-positive nuclei from a total number of 100 basal epidermal cells was determined. Per section, counts were performed in three different locations at a magnification of 200×. Results were expressed as the mean ± SD of the three counts in three donors (error bars).

### RNA isolation and quantitative PCR (qPCR)

At harvesting, the FTMs was cut into small fragments and diluted in a β-mercaptoethanol solution. RNA was isolated from the whole FTM (dermis and epidermis) with the RNEasy Kit (Qiagen, Venlo, The Netherlands). Sufficient amounts of total RNA were available for cDNA synthesis using the iScript™ cDNA Synthesis Kit (Bio-Rad, Veenendaal, The Netherlands) according to the manufacturer’s instructions. Primers (Invitrogen, Breda, The Netherlands) were designed for genes of interest and three reference genes (Table 2). qPCR reactions were performed using the SYBR Green Supermix (Bio-Rad) on the CFX384™ real-time PCR detection system (Bio-Rad). The PCR program included initialisation (6 min at 95 °C), 45 cycles of denaturation (15 s at 95 °C), annealing (30 s at 60 °C) and elongation (30 s at 72 °C), final elongation for 1 min at 72 °C and a DNA melting curve (from 55 °C to 95 °C through 0.2 °C increments every 10 s). All samples were tested in duplicate. Specificity of PCR products was evaluated by size in agarose gel electrophoresis followed by DNA sequence analysis. Serial dilutions of cDNA from spontaneously immortalised keratinocytes (own laboratory) and universal human reference RNA (Stratagene, Santa Clara, CA, USA) were included to determine PCR efficiencies.

**Table 2:** qPCR primers

Gene	Forward primer 5ʹ → 3ʹ	Reverse primer 5ʹ → 3ʹ
Genes of interest		
SERPINB4	CAAAGGGCAGTGGGAGAATA	CCTCCAGCAAGGCAAAATTA
MMP9	CCTGGAGACCTGAGAACCAA	ATTTCGACTCTCCACGCATC
SNAI2	ACAAGCAGCTGCACTGTGAT	ACACAAGGCAATGTGTGGGT
ASMA	GTGTTGCCCCTGAAGAGCAT	GCTGGGACATTGAAAGTCTCA
N-cadherin	TCATTGCCATCCTGCTCTGCAT	AGTTGTTTGGCCTGGCGTTCTT
Reference genes		
RPS29	TATGTGCCGCCAGTGTTTCC	TGCCCCGGATAATCCTCTGA
ZNF410	GCTGTGGTAAGCAGTTTACTACAG	CTTGGGCTTCACAAAGGAAAGG

### Local ethic board approval for the study and informed consent

All primary human skin cells from healthy donors used by the Department of Dermatology are isolated from surplus tissue collected according to article 467 of the Dutch Law on Medical Treatment Agreement and the Code for proper Use of Human Tissue of the Dutch Federation of Biomedical Scientific Societies. According to article 467, surplus tissue can be used if no objection is made by the patient. This means that the patient who will undergo plastic surgery is well informed on the research. In case he/she refuses, the patient has to sign the inform consent form; if they agree, they do not sign. This approach differs from other countries. None of the authors were involved in the tissue sampling and only birth date, gender and skin type of the subjects was known. The Declaration of Helsinki principles were followed when working with human tissue.

## Results

### Fibroblast heterogeneity in SCC tumour stroma

**Fig. 1:**
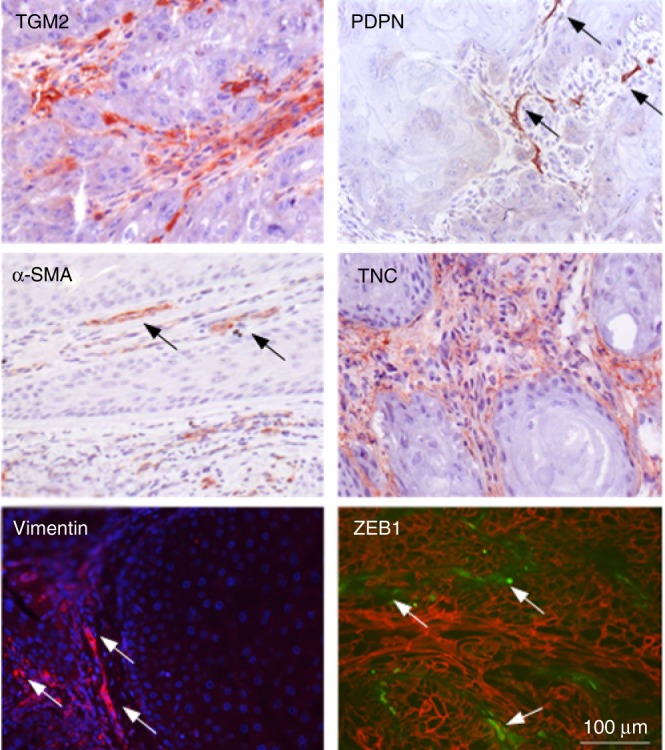
Immunofluorescent and immunohistochemical analysis of vimentin, ZEB1 (green)/β-catenin (red), TGM2, α-SMA, PDPN and TCN expression in SCC biopsy cross sections (*N* = 8) Arrows in PDPN panel point at papillary fibroblasts, those in the alpha-SMA at evident CAFs, and in the vimentin and ZEB1 panels at EMT of tumour cells.

We examined the presence of Pfs and Rfs and EMT-related biomarkers in SCC skin biopsies (*N* = 8). TGM2 for reticular and PDPN and TCN for Pfs were analysed by IHC. As shown in Fig. 1, fibroblasts expressing TGM2 were observed throughout the tumour stroma, while the expression of PDPN and TNC is lower and more restricted. Additionally, we stained for α-SMA, a specific biomarker for myofibroblasts or CAFs, vimentin, a biomarker for mesenchymal cells and ZEB1, a biomarker for EMT. As shown in Fig. 1, a subset of fibroblasts clearly stained positive for α-SMA in the activated stroma, while vimentin and ZEB1 expression was found in the mesenchymal cells surrounding malignant cells.

### In vitro effect of fibroblast subpopulations on SCC invasion

**Fig. 2:**
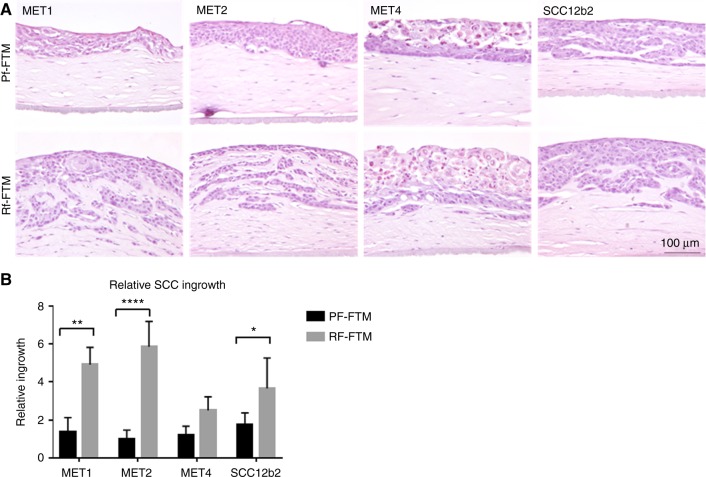
**a** Cross-sections of Pf-FTMs and Rf-FTMs (HE). The epidermis was generated using MET1, MET2, MET4 or SCC12b2 SCC cell lines. **b** Quantification of the ingrowth; the ingrowth is significantly higher in Rf-FTMs of MET1 (*P* < 0.005), MET2 (*P* < 0.0001) and SCC12b2 (*P* < 0.05); *N* = 3

Next we examined the effect of Rfs and Pfs on the invasive behaviour of SCC using FTMs that are generated with a dermal matrix comprising Rfs (Rf-FTMs) or Pfs (Pf-FTMs). To this end, we measured the ingrowth of the different SCC cell lines in HE-stained FTMs (Fig. 2a). As indicated in the figure, in Pf-FTMs onto which different MET cell lines were seeded, no-to-minimal ingrowth was observed in the dermal matrix, while in Rf-FTMs the different MET cell lines had migrated into the dermis. SCC12b2 shows migration in both the papillary and the reticular dermis, but this effect is stronger in the Rf-FTMs as shown in Fig. 2b. Quantification of the migrated areas revealed that the different MET cell lines 1 and 2 and SCCb12 show a significantly higher migration in Rf-FTMs than in Pf-FTMs.

Subsequently, we further characterised these FTMs on epidermal morphogenesis comprising biomarkers for cell activation, BM formation, cell proliferation and differentiation using IHC or immunofluorescence analyses.

**Fig. 3:**
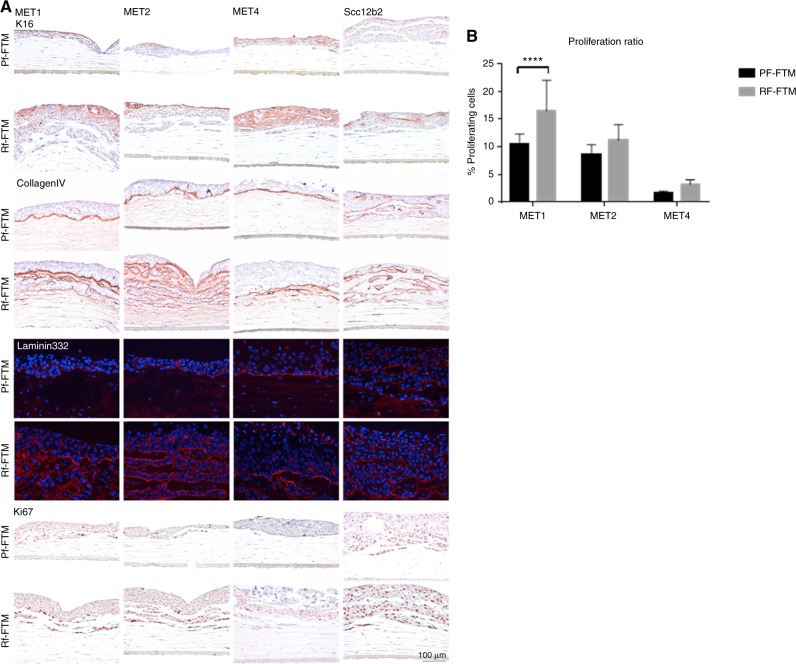
**a** Cross-sections of Pf-FTMs and Rf-FTMs stained for K16, collagen type IV, laminin 332 and Ki67. The epidermis was generated with MET1, MET2, MET4 or SCC12b2 cell lines. Data are obtained for three independent experiments. **b** Quantification of Ki67 in FTMs seeded with MET1, MET2 and MET4. Results are expressed as the mean ± SD of three counts in three donors (*<0.05)

To assess cell activation in Rf-FTMs and Pf-FTMs, we examined K16 (hyperproliferation-specific keratin) expression. As demonstrated in Fig. 3a, K16 is expressed (mostly supra-basal) in all FTMs irrespective of the composition of the dermal matrix, indicating an increased cell activation. However, in Rf-FTMs its expression seems more pronounced. The BM plays an important role in cancer invasion since it acts as a mechanical barrier preventing malignant cells from invading the dermis and deeper tissue. BM formation and integrity was assessed by the expression of ColIV, an important BM protein located at the epidermal–dermal junction (EDJ). In addition, BM homeostasis was assessed by IHC staining of laminin332, appearing as a thin line at the EDJ, indicating the presence of a functional BM (Fig. 3a). In Pf-FTMs, the BM forms a proper barrier between the dermis and the epidermis. However, the Rf-FTMs show disruption of the BM, lining the ingrowth areas in the dermal compartment. Figure 3b shows abundant proliferating cells in the ingrowth areas of the FTMs. By cell count, the percentage of proliferating cells per FTM per area was calculated, which showed more proliferation in the Rf-FTMs compared to that in the Pf-FTMs.

### EMT-related biomarkers abundantly expressed in Rf-FTMs

In response to the clear influence of the fibroblast subpopulations on SCC ingrowth in Rf-FTMs, further experiments were conducted to unravel the underlying mechanism. EMT-specific biomarkers were detected by gene expression and confirmed on protein level.

**Fig. 4:**
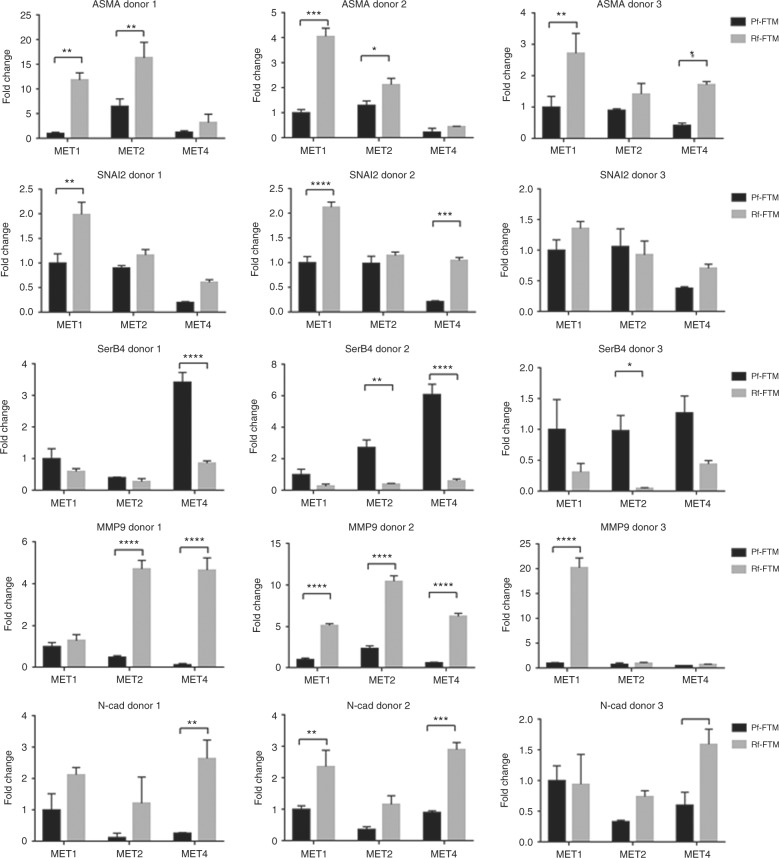
qPCR analysis of the SCC-FTMs for EMT and invasion markers. Error bars represent ±SD *p < 0.05, **p < 0.01, ***p < 0.005, ****p < 0.0001

Gene expression analysis revealed clear trends in SCC-FTMs derived from three Rf and Pf donors, shown in Figure 4. α-SMA, SNAI2 and N-cadherin, three essential biomarkers for EMT, were overexpressed in Rf-FTMs. Also, matrix metalloproteinase 9 (MMP9), a metalloproteinase responsible for breakdown of the BM in tumour invasion, is strongly upregulated in the Rf-FTMs. However SerpinB4, an important SCC biomarker, is more highly expressed in the Pf-FTMs.

**Fig. 5:**
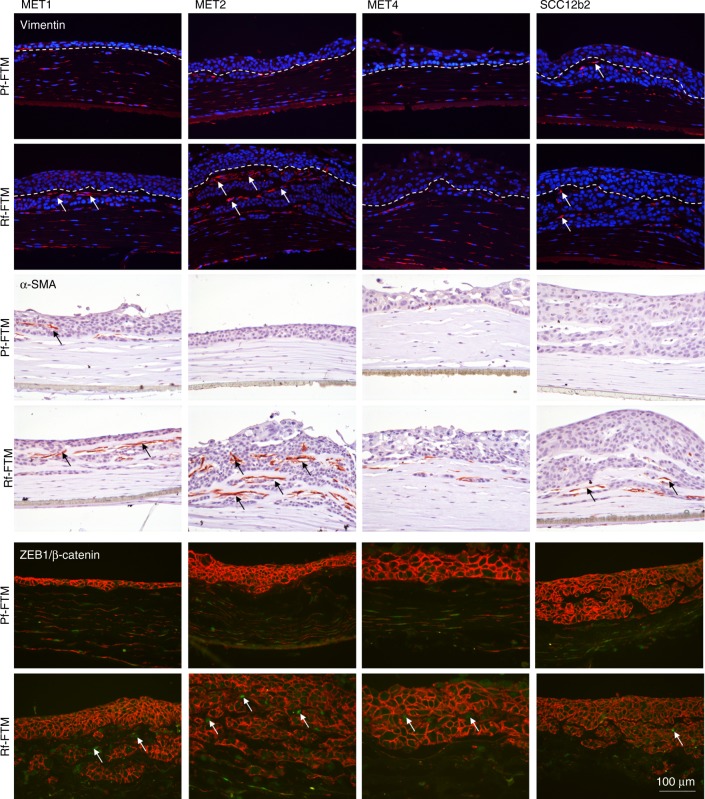
Shown are cross-sections of Pf-FTMs and Rf-FTMs that were stained for vimentin, α-SMA and ZEB/β-catenin. The epidermis was generated with MET1, MET2, MET4 or SCC12b2 cell lines. Arrows indicate the expression of these biomarkers surrounding the SCC ingrowth. Data are obtained for three independent experiments

Increased vimentin expression is frequently used as an EMT marker in cancer. In Fig. 5, vimentin is visible in both fibroblasts types and clearly in cells between the epithelium and the ingrowth areas, as indicated by the arrows. An increased expression in the Rf-FTM is visible, with the strongest signal in the MET1 and MET2 Rf-FTMs. At this exact same localisation α-SMA is expressed. α-SMA is both an important EMT and CAF marker found in the reactive tumour stroma. The increased α-SMA gene expression in Rf-FTMs is hereby confirmed on the protein level.

This correlates to the expression of ZEB1, also shown in Fig. 5. Transcription factor ZEB1 induces EMT by downregulating E-cadherin and upregulating a number of other mesenchymal markers, e.g., vimentin, thereby facilitating cell migration and invasion. More ZEB1 expression is visible in the Rf-FTMs, especially in MET1 and MET2 SCC cells appearing at the same localisation as the vimentin- and α-SMA-positive cells.

## Discussion

### Characterisation of SCC in vitro and effects of fibroblast subpopulations on SCC progression

Examination of the tumour stroma in SCC biopsies showed a dynamic and diverse landscape of fibroblast lineages surrounding malignant cells. By insulating Pfs and Rfs in the in vitro SCC-FTMs, we investigated the role of fibroblast subtypes in the tumour microenvironment on SCC behaviour and possibly EMT-like mechanisms. In this study, it is demonstrated for the first time that Pf and Rf subtypes strongly influence the tumour stroma and thereby contribute to SCC invasion. Pfs contain the cancer cells within the epidermis, while Rfs are clearly predisposed to differentiate into CAFs upon SCC signals, assisting invasion and initiating EMT. We reconfirmed these observations in another set of experiments in which we mixed the different SCC cell lines with NHEKs (ratio 1:4) and evaluated the expression of the early (K10) and late differentiation (loricrin) proteins in both FTM models (supplementary figure 1). The tumour stroma is an integral part of cancer progression.^23^ Individual components of the stroma, in particular CAFs, play an important role in these processes.^24^ Insights have emerged regarding communication between fibroblasts and epidermal cells.^25^ Signalling molecules, released by fibroblasts, influence keratinocyte migration, proliferation and differentiation, all of which are key elements in SCC development.^26–28^ This communication between malignant cells and their environment is modelled and demonstrated in vitro.

### FTMs mimic SCC behaviour

The effect of the Pf and Rf subtypes on SCC ingrowth was striking. The measurements showed significantly more migration of malignant cells into the dermis in Rf-FTMs compared to that in Pf-FTMs. In this study, the models are examined for their SCC characteristics, and further on, the mechanism behind this phenomenon is unravelled. In healthy skin, proliferation and differentiation of epidermal keratinocytes is tightly controlled to ensure proper development and homeostasis of the epidermis. In SCC, homeostasis is completely disrupted. This phenomenon is also observed in our FTMs that were generated with a reticular dermis.

In the epidermis, K16 and K17 expression is absent under normal conditions. K16 and K17 are induced during wound healing where the skin shows hyperproliferation and aberrant differentiation.^29,30^ This induction correlates with the onset of keratinocyte activation, and therefore, it is hypothesised that these keratins are involved in the process of SCC.^31,32^ In a previous study from our group, we have shown that K16 and K17 were listed in the top three genes that was significantly upregulated in actinic keratosis and SCCs and could hereby be used as a biomarker for this epithelial cancer.^33^ Expression of both K16 and K17 (data not shown) was more pronounced in Rf-FTMs generated with MET 1 and 4 when compared to Pf-FTMs. One may speculate that this difference may be explained by the different stages of the SCC cell lines. Malignant cells often harbour an increased cell proliferation. Detection of the cell‐cycling-specific marker Ki67 showed more cell proliferation in the reticular models of MET1, MET2 and MET4. With these findings, we can confirm that the cell lines show typical SCC progression on FTMs, but this phenotype is more pronounced in the Rf-FTMs.

The BM is the first and most robust structural barrier to invasion.^34^ In the normal skin, the BM separates epithelium from the underlying connective tissue, the dermis. After destruction of the BM, tumour cells invade locally or metastasise to distant sites.^14^ To evaluate BM formation, expression of collagen type IV and laminin332 in FTMs was assessed. Many of the key elements of the BM, including collagen type IV, have been shown to be dysregulated in SCC.^35^ In conventional FTMs, the BM is formed in a comparable way as in the in vivo situation and shares the same characteristics. In the Pf-FTMs, collagen type IV and laminin332 were evenly distributed along the BM, separating the dermis from the epidermis. In the reticular models, we noticed a disruption of the BM, framing the ingrowth areas in the dermis, where we observe cell islands. However, when modelling invasion, the BM should be first fully formed and functional before any invasion in underlying connective tissue may proceed.^22^

### Rf-FTMs are prone to EMT

A crucial set of EMT markers was selected and evaluated at the gene and protein expression level. Gene expression analysis showed an overexpression of certain EMT biomarkers among all donors. Transcription factors responsible for EMT are ZEB1, SNAI and TWIST.^36^ An overexpression of SNAI2 is shown in Rf-FTMs, together with an upregulation of the cell-surface protein N-cadherin, which correlates with high risk of SCC invasion via EMT-like mechanisms.^37^ In SCC, the tumour tissue is surrounded by reactive stroma, made up mostly of CAFs, known for their expression of α-SMA. This expression is related to the stage of tumour development and patient prognosis.^38^ In Rf-FTMs, a strong upregulation (2–15-fold) of α-SMA mRNA is shown; in further analysis, this upregulation is confirmed at the protein level by IHC. The fibroblasts in Rf-FTMs express α-SMA when they are localised near the malignant SCC cells, meaning activation of the stroma. Cells positive for α-SMA contribute to EMT by their increased contractile mobility and ECM remodelling ability.^3^ Importantly, since in normal Pf-FTMs or Rf-FTMs no cells stain positive for α-SMA (data not shown), one may infer that Rfs differentiate into CAFs due to factors secreted by the MET cell lines. This hypothesis is strengthened by an increased expression of vimentin and ZEB1 at the same spots as the α-SMA-positive cells, indicating signalling between the SCC cells and their surrounding stroma. Cytoskeletal protein vimentin is used to distinguish mesenchymal cells from epithelial cells. It is abundantly expressed in fibroblasts. When cells undergo EMT, they adopt a mesenchymal phenotype where vimentin is expressed at the sites of cellular elongation.^39^ Observed ZEB1 contributes to malignant progression by upregulation of N-cadherin, vimentin and MMPs and is therefore an important EMT inducer.^15^

Among the most commonly identified metalloproteinases in SCC is MMP9.^40,41^ MMP9 is a gelatinase, which degrades collagen type IV, most critical in degradation of the BM. We show upregulation of MMP9 in Rf-FTMs, compared to Pf-FTMs. MMPs were initially thought to be produced solely by the tumour cell, but further investigation has also shown production by CAFs and the surrounding inflammatory cells.^41^ Recently, it has been shown that MMP9 could even lead to activation of EMT genes.^42,43^ In this way, it is plausible that the tumour stroma consisting of Rfs is involved in MMP9 upregulation and facilitate SCC invasion via EMT.

SerpinB4 is described as a biomarker of cancer and known to be strongly upregulated in SCC indicative of a poor prognosis.^44,45^ Although the process is not yet fully understood, SerpinB4 also acts as an EMT promoter.^46^ It is expressed by both epithelial cells and fibroblasts upon oncogenic Ras activation.^47^ Interestingly, in this study SerpinB4 is strongly upregulated in the Pf-FTMs. This seems inconsistent with the literature so far. However, it could also be one of the important features of the possible protective function of the papillary dermis that may give new insights in the influences on the tumour stroma.

In this study, we show for the first time that a functional link exists between the two fibroblasts subtypes in the dermis and invasive behaviour of SCC. Rfs and Pfs create distinct tumour microenvironments that differently affect SCC progression. Where the reticular dermis forms a tumour-promoting environment, the papillary dermis protects against tumour invasion. Pfs can differentiate into Rfs after in vitro ageing (prolonged culture) and Rfs could then be the precursors of CAFs since they are predisposed to differentiate or be 'activated' after interaction with the tumour.^12^ This differentiation or 'activation' process may be also valid for different fibroblast subtypes with other types of epithelial cancers.

## Electronic supplementary material

Supplementary data
