# Supplementary material for: A shift from papillary to reticular fibroblasts enables tumour–stroma interaction and invasion
Source: Br J Cancer. 2018 Mar 19;118(8):1089–97. doi: 10.1038/s41416-018-0024-y (PMC5931114; doi:10.1038/s41416-018-0024-y)
Supplement: Supplementary file 1 — Supplementary data [file 41416_2018_24_MOESM1_ESM.docx]

# Supplementary data

## Keratinocyte differentiation in SCC-keratinocyte generated FTMs


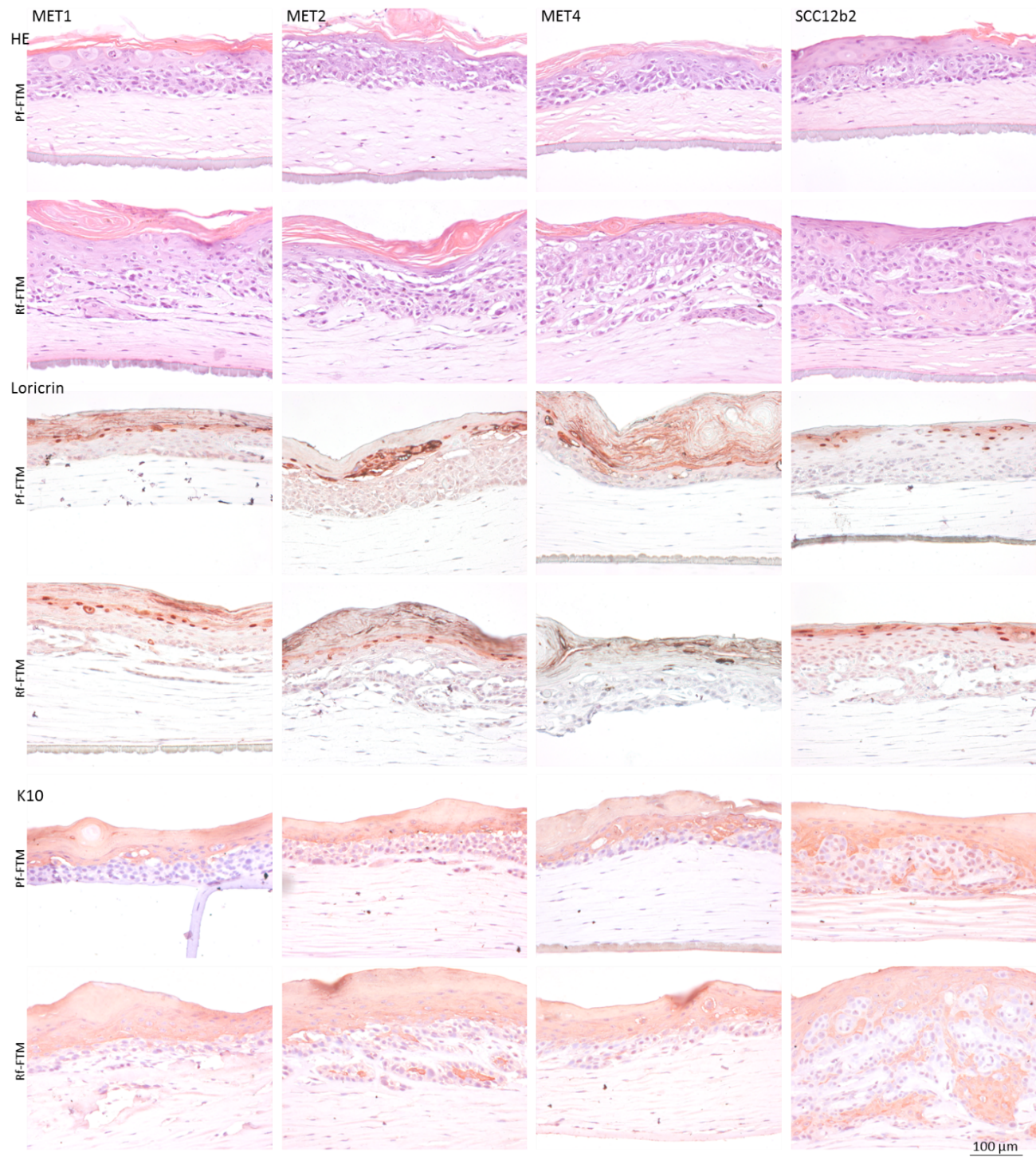


Figure 1: Shown are cross-sections of Pf-FTMs and Rf-FTMs that were examined for general morphology (HE) and epidermal differentiation (K10 and loricrin). The epidermis was generated with primary human keratinocytes mixed with MET1, MET2, MET4 or SCC12b2 or SCC cell lines, respectively.

In healthy skin, keratinocytes differentiate and move upwards while they cornify into the stratum corneum . This process takes about 1 month. *In vivo*, SCCs show less differentiation; the SCC cells lost their ability to differentiate and their polarity to move upwards. However, the stratum corneum is still formed. But SCC-FTMs show no signs of keratinocyte differentiation as demonstrated in figure 2A.

Therefore our second aim was to generate a new FTM that mimic the *in vivo* situation. For this purpose SCC cells and healthy keratinocytes were co-cultured, in the same way as they coexist in the *in vivo* situation. The results of these new FTMs are shown in the following section of this report. The HE staining (Figure 6) of the co-cultured FTMs shows exactly what was expected. They formed a nicely differentiated epidermis and a stratum corneum, the same ingrowth pattern as in the single cultures, and a minimal ingrowth in the papillary model and clearly more ingrowth in the reticular models.

To further asses the differentiation stages of the FTMs, a k10 and loricrin staining was performed. K10, an early differentiation marker, is normally expressed in all suprabasal layers. Also co-cultured FTMs all show terminal differentiation by expressing (late differentiation marker) loricrin in the most outer layer of the epidermis. This correlates with the fact that there was a visible stratum corneum in the HE staining.
